# Stability of Expanded Austenite During Annealing in Vacuum

**DOI:** 10.3390/ma18030546

**Published:** 2025-01-25

**Authors:** Stephan Mändl, Hyemin Oh, Daniel Hristov, Darina Manova

**Affiliations:** Leibniz Institute of Surface Engineering (IOM), Permoserstr. 15, 04318 Leipzig, Germanydarina.manova@iom-leipzig.de (D.M.)

**Keywords:** expanded austenite, stainless steel, CrN precipitates, in situ XRD, ToF-SIMS

## Abstract

In situ X-ray diffraction has been used to investigate the stability of expanded austenite during annealing in vacuum for the austenitic stainless steel 316Ti, the super-austenitic stainless steel 904L, and the duplex steel 318LN. Expanded austenite has been formed using plasma immersion ion nitriding before. Time-of-flight secondary ion mass spectrometry before and after annealing yielded complementary information regarding nitrogen depth profiles and CrN precipitation using cluster analysis. The decay of expanded austenite during annealing was found to be thermally activated with an activation energy of 1.8 ± 0.3 eV, starting within five minutes at 550 °C and taking more than two hours below 450 °C. The decay occurs simultaneously throughout the whole nitrogen-containing zone—and not at the surface as during nitriding. Nitrogen diffusion occurring in parallel slightly complicates the data analysis. Further transmission electron microscopy investigations are necessary to understand the microstructure after annealing in vacuum. The limit for operating hard and wear-resistant expanded austenite layers at elevated temperatures of up to 350 °C is given, however, by nitrogen diffusion and not the decay into CrN.

## 1. Introduction

Austenitic stainless steel has been developed as a material with a very high corrosion resistance compared to low alloy steel. However, it is a rather soft material and prone to high wear rates and a tendency towards cold-welding when operated without lubrication. Hence, surface nitriding has been developed with the aim of increasing its hardness together with a strongly reduced wear rate [1,2,3]. Thus, nitrogen insertion in the temperature range between 300 and 450 °C leads to a super-saturated phase expanded austenite where nitrogen is still in solid solution [4,5,6,7]. A maximum nitrogen content of 38 at.% results in an anisotropic lattice expansion of up to 12% [8].

Nevertheless, it is a metastable phase, and it decays into CrN or Cr_2_N and a nitrogen-free Fe-Ni phase [9]. During nitriding, this decay limits the allowed processing time to a few minutes at 500 °C, increasing (theoretically) to thousands of hours at 300 °C [10]. Similarly, carbon-expanded austenite formed after carburizing has an incubation time of thousands of hours at 350 °C during carburizing [11]. While this mixed phase is still very hard and wear-resistant its corrosion protection is highly compromised as no protective chromium oxide layer can dynamically be formed at the surface [12,13].

This decay of expanded austenite induced by the formation of CrN precipitates has been described in the literature during nitriding for higher temperatures and a timescale measured in hours [1,5,11]. Nevertheless, it is also of interest what happens after nitriding during the service life: Is this transition without a continuous supply of nitrogen identical or different from the situation during nitriding taking place? Can expanded austenite be used at slightly elevated temperatures for long periods of time? As expanded austenite is also observed for nitriding of super austenitic [14,15] and duplex stainless steel [16,17], it is also interesting to analyze what happens there. While experiments for several thousands of hours are very time-consuming, as a first step annealing in vacuum at temperatures similar to those used for nitriding can be investigated. Here, the influence of the nitriding temperature and the alloy composition is investigated using in situ X-ray diffraction (XRD) during annealing.

As expanded austenite is a well-investigated material, a direct extraction of the local nitrogen content—in the expanded austenite—from the lattice expansion is possible [18]. At the same time, cluster analysis in time-of-flight secondary ion mass spectrometry (ToF-SIMS) allows us to determine the total nitrogen content and the existence of CrN precipitates at a certain depth. Hence, two different methods—XRD and SIMS, as an alternative to a more complex and time-consuming analysis using transmission electron microscopy (TEM)—are available to examine the local phase composition and microstructure of the stainless steel samples.

Accordingly, the current manuscript compares the behavior of different types of stainless steel after nitriding during annealing in vacuum. The focus is on the stability of the expanded austenite against decay into CrN precipitates and an Fe-Ni-rich phase. The diffusion of nitrogen towards the bulk, and thus the increasing layer thickness, is a competing process.

## 2. Materials and Methods

Three different stainless steels, the austenite AISI 316Ti (X6CrNiMoTi17-12-2, equivalent to DIN 1.4571, according to DIN/EN [19] (in wt.%) C ≤ 0.08, Cr 16.5–18.5, Ni 10.5–13.5, Mo 2.0–2.5, Ti 5 × C ≤ 0.70), the super austenite AISI 904L (X1NiCrMoCu25.20.5–DIN 1.4539 with C ≤ 0.02, Cr 19.0–21.0, Ni 24.0–26.0, Mo 4.0–5.0, Cu 1.2–2.0), and the duplex steel AISI 318LN (X2CrNiMo22.5.3–DIN 1.4462 with C ≤ 0.03, Cr 21.0–23.0, Ni 4.5–6.5, Mo 2.5–3.5) were used. Coupons were cut from cylindrical rods with a thickness of 2.5 mm, ground, and polished up to a mirror finish.

These three alloys were chosen to cover three important groups of stainless steel (austenite, super austenite, and duplex) while being able to compare the results during annealing with existing literature on nitriding. While there are many more alloys, these are commonly used in industry as well as for nitriding experiments in the laboratory.

The nitrogen plasma immersion ion implantation process was performed in a UHV chamber using an electron cyclotron resonance plasma source operating at 150 W at a nitrogen background pressure of 0.6 Pa [20]. Pulses of 7 kV with a length of 15 µs were applied to the substrate holder with the initial frequency for the heating phase at 5 kHz. After reaching 450 °C in less than 15 min, a frequency of 1.65 kHz was enough to sustain the temperature by the energetic ion bombardment for the rest of the nitriding process (for 500 °C, a slightly higher frequency of 2.1 kHz was maintained). Table 1 summarizes the nitriding conditions.

The annealing experiments were performed in an HV vacuum system used for in situ XRD measurements with the chamber attached to a diffractometer in Bragg–Brentano geometry together with an external sample heating system. The sample temperature maintained constant via a fast control loop, is measured during the whole experiment with a thermocouple located very close to the sample surface. More details on the experimental equipment can be found in ref. [21]. At the same time, the narrow substrate peaks observed in XRD for stainless steel can be used for measuring the temperature independent from the thermocouple with a resolution of better than 5 K [21]. The heating phase lasted typically 20 min to reach 400 °C. Annealing was then performed at constant temperatures, varying between 370 and 550 °C, for up to two hours.

The acquisition time for one diffractogram encompassing a 2θ range from 35 to 54 deg is slightly less than 2 min as a position-sensitive linear X-ray detector is used. The wavelength of 0.15418 nm corresponds to Cu Kα radiation. The known attenuation length [22] corresponds to an information depth of around 2.5 µm for the current angular range. Thus, the collected signals can originate anywhere in this depth; however, the near-surface region is the dominant source. Information from deeper regions is not accessible in this way.

Dynamic ToF-SIMS measurements (IONTOF SIMS V, Münster, Germany) were carried out in a dual beam configuration with 15 keV ^69^Ga^+^ ions–2 pA–for the analysis and 2 keV O_2_^+^ ions–700 nA–for depth-profiling. The respective scan areas were chosen to be 100 × 100 μm^2^ and 300 × 300 μm^2^, thus avoiding crater edge effects for the analysis beam. The resulting sputter rate was about 0.8 nm/s for the employed scan size and ion current. This rate was determined from the final crater depth under the assumption of a sputter rate, which was constant with time and independent of the actual chemical composition of the surface layer. Absolute calibration of the count rate in SIMS for carbon and nitrogen was obtained by comparing the SIMS data of a reference sample with glow discharge optical emission spectroscopy (GDOES) by a company (TAZ GmbH, Aichach, Germany, accreditation according to [23]—a Europewide quality standard for analytical testing laboratories).

## 3. Results

**Figure 1:**
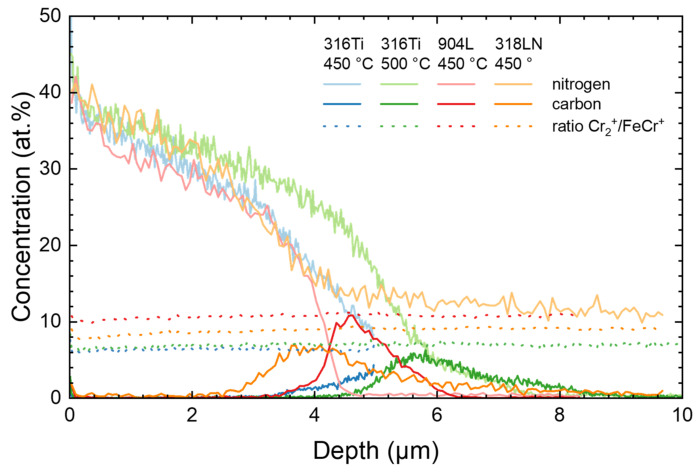
SIMS depth profiles after nitriding using the four different materials and treatment combinations detailed in Table 1. Next to carbon and nitrogen, the cluster ratio is plotted to yield information on the CrN precipitation [27].

Before discussing the behavior of the different steel grades during annealing in vacuum, the expanded austenite formed during nitriding was characterized. Figure 1 presents the SIMS data obtained for these steel samples after nitriding. Regarding the nitrogen content, the typical depth profiles known for expanded austenite [15] are obtained with a slow decrease in the plateau-like region near the surface, followed by a rather sharp decay near the interface. The results are in good agreement with the very sharp transition between expanded austenite and the substrate observed in SEM cross sections for the nitrided samples [20,24] when considering the increasing roughness inside the sputter crater that induces a broadening of the profiles with depth [25]. As a certain amount of nitrogen (0.10–0.22 wt% according to the DIN standard [19]) is already present in 318LN, the concentration does not reach zero beyond the zone containing the expanded austenite, in contrast to the three other samples.

Carbon occupies the same interstitial sites as nitrogen while it diffuses faster than nitrogen; hence, the originally present carbon is agglomerating at the interface just below the nitrogen. This behavior is well known in the literature [26]. When looking at the Cr_2_^+^/FeCr^+^ ratio, it is constant throughout the layer of expanded austenite and the substrate. Previous investigations have shown that here no CrN precipitation has occurred during nitriding [27]. While for 450 °C this can be expected, whereas at 500 °C and 60 min; it is, in contrast, close to the limit where the transition should start [28]. However, a very thin layer of CrN precipitates of less than 25 nm at the surface cannot be ruled out with the resolution and sensitivity of the current data set.

**Figure 2:**
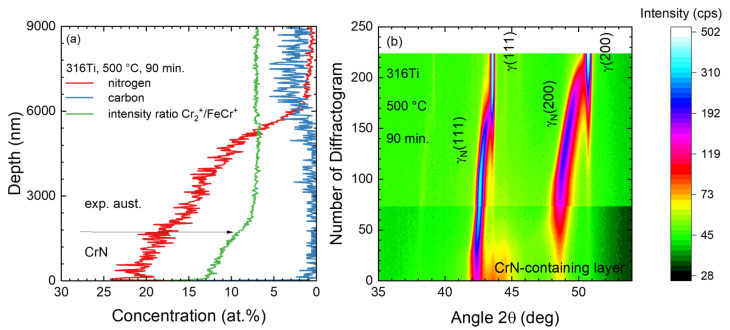
Comparison of (**a**) SIMS profiles and (**b**) 2D-contour plots of X-ray diffractograms for a 316Ti sample nitrided at 500 °C for 90 min. The in situ data were taken during sputter etching. Thus, the number of diffractograms corresponds to a depth scale for the image (reproduced from S. Mändl and D. Manova, Metals; published by MDPI, 2024 [15]).

For comparison, the data in the literature showing depth profiles from ref. [15] are replotted in Figure 2 where 316Ti has been nitrided for 90 min. at 500 °C. There, the CrN formation near the surface is clearly visible in both the SIMS depth profiles as well as the contour plot of diffractograms measured during the sputter etching of this sample. In SIMS (Figure 2a), the transition from CrN to expanded austenite can be seen in the change in the cluster ion ratio Cr_2_^+^/FeCr^+^. In the XRD contour plot (Figure 2b), these two phases give rise to separate reflections. In general, this layered structure representing a transition stage during the decay of expanded austenite is also visible in SEM cross sections [29].

**Figure 3:**
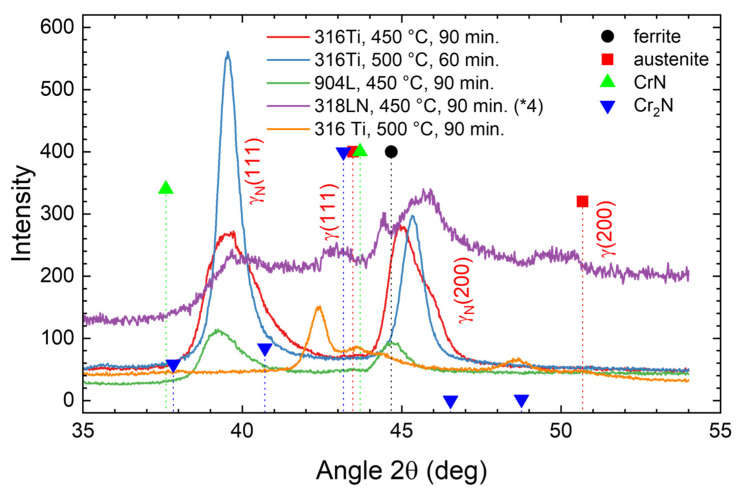
XRD diffractograms after nitriding before annealing. Please note that the data for the sample 318LN are multiplied by a factor of four to improve the readability. “×4” indicates that the intensity was multiplied by a factor of 4 before plotting the data.

The XRD diffractograms of the four samples depicted in Figure 1 are plotted in Figure 3 as additional information on the nitrided samples before the annealing step, together with the data obtained for the CrN-containing sample shown in Figure 2 (316 Ti, 500 °C, 90 min—which is not used for the annealing experiments below). For the four nitrided samples without CrN precipitation, the expanded austenite phase is visible with rather broad and asymmetric peaks (γ_N_(111) and γ_N_(200)) shifted noticeably towards the left of the austenitic substrate peaks. The lattice expansion of about 7–10% is higher for the (200) oriented grains than for the (111) oriented grains (always measured normal to the surface). The peak width is roughly determined by the gradient of the nitrogen concentration with steeper gradients leading to broader peaks as the X-ray intensity from the diffraction from deeper regions is attenuated by the transit through the overlying material [22].

For the duplex steel, rather low intensities are observed with a reduced signal-to-noise ratio; there, the substrate is barely visible. For the sample nitrided at 500 °C for 90 min used as a reference only, a mixture of expanded austenite, austenite, and CrN is observed, due to the layered structure shown in Figure 2. Thus, the XRD data, keeping in mind the finite information depth of the method, are in broad agreement with those to be expected from the nitrogen depth profiles observed with SIMS, leading to the formation of expanded austenite near the surface and its eventual decay.

After these precluding remarks, the stability of the expanded austenite formed by nitriding is investigated separately for the four nitrided samples. In the first subsection, the data for the alloy 316Ti, nitrided at two different temperatures and annealed at a range of temperatures are presented.

### 3.1. Stability of Expanded Austenite in 316Ti

**Figure 4:**
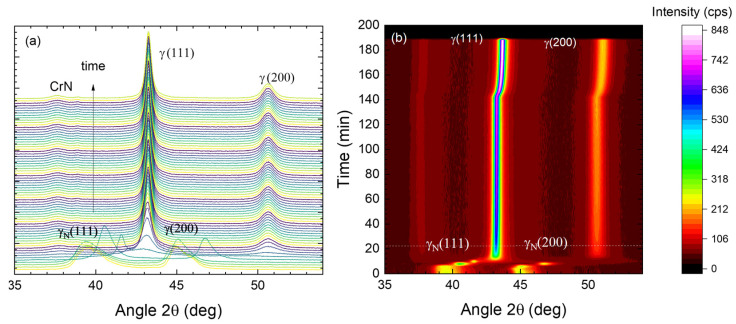
Diffractograms of 316Ti, nitrided at 450 °C, during annealing at 550 °C: (**a**) time series (the colors are just used to help distinguishing between the separate diffractograms; (**b**) 2D-contour plot of the identical data. One diffractogram was measured every two minutes; thus, 200 min corresponds to 100 diffractograms.

Figure 4 presents the diffractograms obtained for 316Ti, previously nitrided at 450 °C, during annealing in vacuum at 550 °C. In the left panel (a), the measured diffractograms are plotted, and vertically shifted for clarity, whereas the right panel (b) shows the 2D-contour plot of the same data. While more details of the diffractograms, especially peak width and line shape are visible on the left, the right panel gives a better overview of the total data. At the beginning, the heating phase of about 8 min can be identified; there, the γ_N_(111) and γ_N_(200) peaks are slightly shifting towards the left (due to the thermal expansion of the steel). After reaching 550 °C, a very rapid decay of expanded austenite within the next 5 min is observed, followed by grain growth (visible in the reduced peak width up to 50 min). At the end beyond 145 min, the slow cooling phase is similarly visible with a kink occurring for the then visible γ(111) and γ(200) peaks.

**Figure 5:**
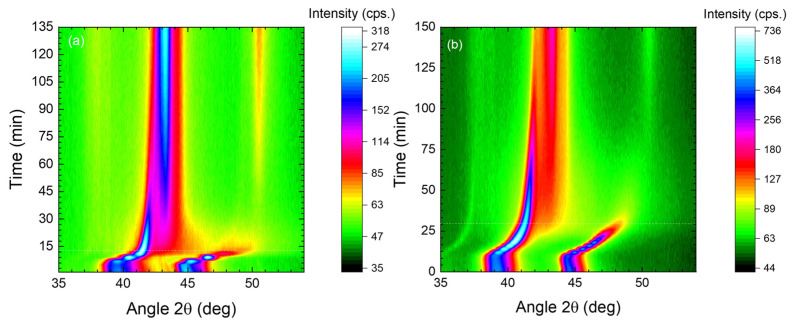
Two-dimensional contour plots of 316Ti, nitrided at 450 °C, during annealing at (**a**) 500 °C and (**b**) 450 °C.

While no peaks corresponding to CrN are visible, this observed behavior is typical for the decay of expanded austenite into nanocrystalline CrN precipitates and a Fe-Ni-rich matrix: as a gamma phase and not a ferritic phase, which should be favored as the Cr depletion changes the stability of the fcc phase [12]. As the transition occurs immediately after the heating to 550 °C, no detailed time evolution can be deciphered. For slightly lower temperatures of 500 and 450 °C (with the 2D-contour plots shown in Figure 5), the expanded austenite is successively more stable, and the decay starts later. Nevertheless, even at 450 °C, after less than 30 min at this temperature, no expanded austenite remains.

**Figure 6:**
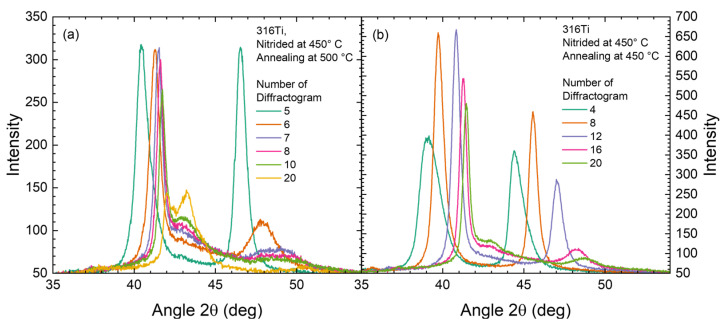
Selected diffractograms from Figure 5: (**a**) 500 °C annealing, (**b**) 450 °C annealing.

For a more detailed view of the period where the most prominent changes occur with time in Figure 5, selected diffractograms of the time series for these two samples are shown in Figure 6. At 500 °C (cf. Figure 6a), a nearly instantaneous transition from the expanded austenite towards the decayed structure is observed at the beginning between diffractograms #6 and #8. In contrast, at 450 °C this phase decomposition is delayed and starts only between diffractograms #12 and #16. In both cases, the co-existence of expanded austenite and the decayed structure is not observed. Neither are two peaks simultaneously visible nor is the shape or peak width of the expanded peak modified—which would be expected if the surface region with a higher nitrogen content is suddenly changed to a different phase. Thus, despite an information depth of about 2.5 µm, no transition front moving through the expanded austenite (in contrast to the behavior observed for the decay during nitriding [15,29]) is noticeable. Thus, the transition appears to start throughout the layer simultaneously.

Another effect to be mentioned is the change in the expanded phase at 450 °C before CrN precipitation starts: as can be seen in Figure 6, the lattice expansion is reduced, and the peaks are becoming narrower. Nitrogen is mobile in expanded austenite (to a certain degree, depending on the specifics of the respective trapping/detrapping model [30,31]). Hence, first, the local nitrogen content, near the surface as probed by XRD, is decreasing and, second, the concentration gradient between the expanded phase and the substrate is gradually reduced as the layer thickness increases. These two effects can explain the change in the positions of the reflections of the expanded austenite towards larger angles. While this change in concentration should be dominant, minor contributions from stress relaxation during annealing cannot be ruled out [1,24,32].

**Figure 7:**
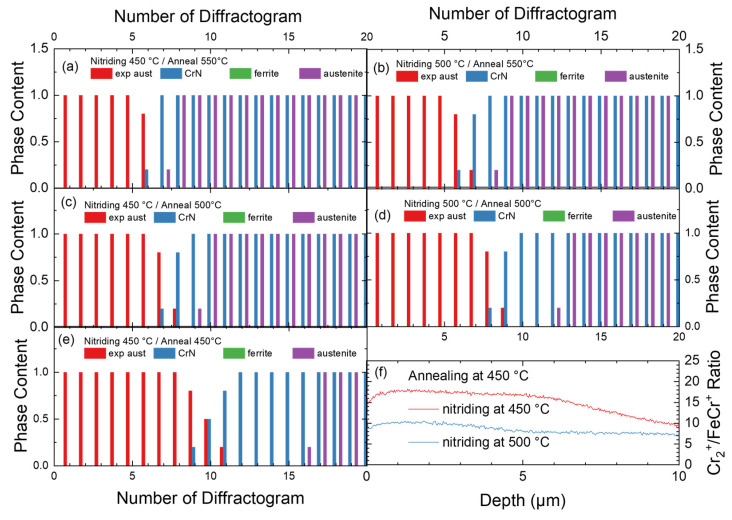
(**a**–**e**) Bar graphs of the phase composition as a function of time and annealing temperature for three different annealing temperatures and two nitriding conditions for 316Ti. The heating phase is included in the time series. Please note that no ferrite was observed. (**f**) Comparison of the Cr_2_^+^/FeCr^+^ ratio in SIMS for annealing at 450 °C.

As the decay is time-dependent, it is possible to plot the corresponding evolution of the phase transition during the annealing at higher temperatures as a function of time. For all conditions where the decay of expanded austenite and thus CrN formation was observed, the bar graphs of the qualitative phase composition are shown in Figure 7. For annealing at 550 and 500 °C, both samples nitrided at different temperatures show the decay of expanded austenite, whereas for annealing at 450 °C only nitriding at 450 °C leads to the decay. In contrast, after nitriding at 500 °C, annealing at 450 °C does not modify the phase composition.

Ex situ SIMS data confirm the phase transformation observed in in situ XRD indirectly using the cluster analysis, as shown in the comparison for annealing at 450 °C in Figure 7f. There, annealing leads to a similar layer thickness of around 10 µm, yet CrN is only marginally present within the first 4 µm for the sample nitrided at 500 °C after annealing at 450 °C. While the first 3–4 µm from the surface seem to contain some CrN precipitates up to 5–10%, this fraction is too small to become visible in XRD. Neither the expected reduction in the intensity of the expanded austenite reflections nor additional CrN reflections with an estimated grain size of around 10–25 nm are reflected in the diffractograms [27]. In contrast, the complete layer consists of CrN when nitrided at 450 °C and annealed at 450 °C (the nitrogen depth profile is shown below).

**Figure 8:**
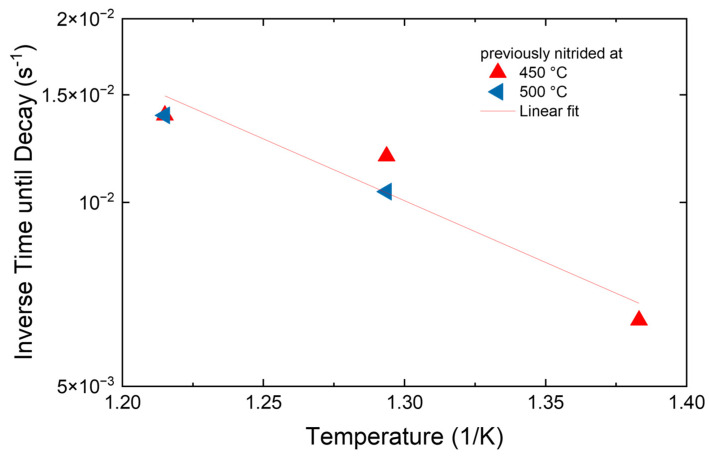
Arrhenius plot of the decay of expanded austenite as a function of the inverse temperature. The calculated activation energy is 1.8 ± 0.3 eV.

As a next step, the time needed to start the transition (more exactly, the inverse time) is plotted vs. the inverse temperature in an Arrhenius plot to find the thermal activation energy as shown in Figure 8. Unfortunately, only three temperatures are available with the data points for 550 °C already very close to the time resolution of the XRD experiment. Nevertheless, the value of 1.8 ± 0.3 eV for the activation energy is in reasonable agreement with the data in the literature on the stability of expanded austenite during nitriding [28,33]. However, these data for the decay obtained during nitriding compress two different processes—local nucleation and propagation of the decay zone within one activation energy [27,29]. At the same time, no comparison of absolute timescales for the transformations is possible between the current experiment during annealing in vacuum and the data in the literature for the decay during nitriding. Here, a separate experiment with low-temperature nitriding followed by immediate high-temperature nitriding could be helpful in identifying nucleation centers for the CrN precipitation.

This experimental derivation of the activation energy for the decay of expanded austenite during annealing in a vacuum is the major result of the in situ data. However, additional information on diffusion processes and the possible distribution of nitrogen inside the lattice can be derived when looking at the peak position of the expanded austenite reflections as a function of time.

**Figure 9:**
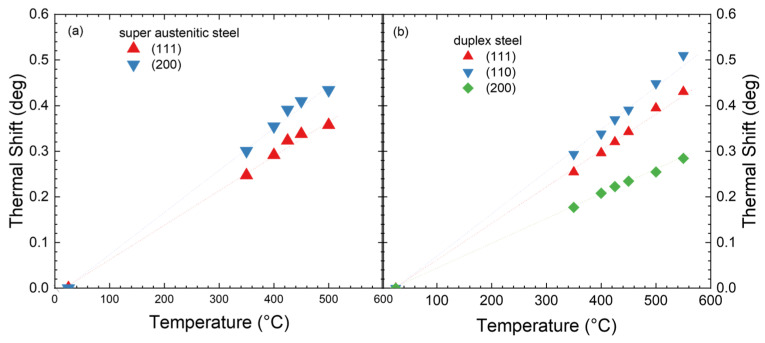
Thermal shift for the XRD reflections for (**a**) super austenitic and (**b**) duplex steel. For austenitic stainless steel, a behavior very close to that for super austenitic steel was observed.

However, before determining the nitrogen content from the lattice expansion, it must be corrected for thermal expansion. This leads to an additional shift towards smaller angles, similar to and overlapping with the effect of nitrogen insertion. As there are nearly no data available in the literature, and the thermal expansion may vary for different classes of alloys, the in situ XRD equipment was used to measure the thermal shift for the alloys of austenite, super austenite, and duplex steel used in the current experiment. As shown in Figure 9a, a roughly linear shift in the diffraction angle (which is more relevant for the current discussion than the linear expansion coefficient) is observed for super austenitic steel 904L (the data for austenitic steel 316Ti are very similar). For duplex steel, slightly different data are measured and separated for the ferritic and the austenitic components. While the measurements are only for lattice planes normal to the surface, an isotropic thermal expansion can be assumed.

**Figure 10:**
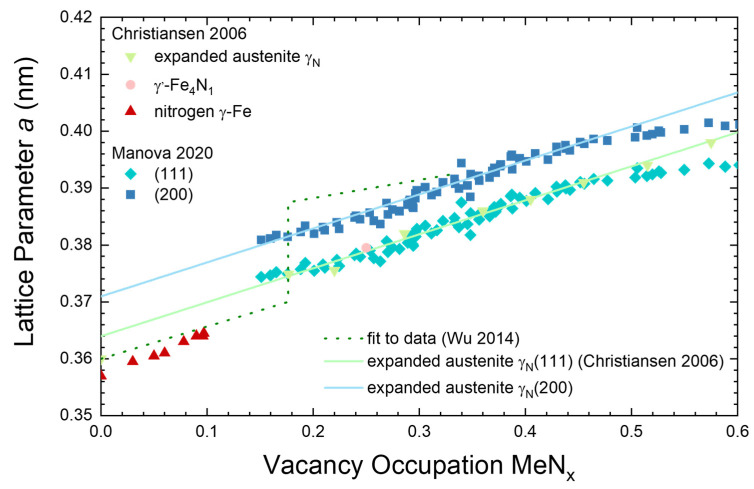
Lattice parameter *a* as a function of occupation of the vacancy sites with nitrogen from the data in the literature. The linear interpolation used in the manuscript for the two orientations is shown as solid lines. Adapted and reprinted from *Surf. Coat. Technol. 456*, D. Manova, S. Mändl, Initial phase formation during nitriding of austenitic stainless steel, 129258, Copyright 2023, with permission from Elsevier [35]. Christiansen 2006 [18], Manova 2020 [36], Wu 2014 [37].

Usually, Vegard’s law is invoked when determining the lattice constant for a series of compositions in a binary alloy system. For austenitic stainless steel, there are published values for the lattice constant as a function of nitrogen content [24]: *a*(γ_N_) (nm) = 0.3587 + 9 × 10^−4^ × *C*_N_ (at.%). However, these values are valid only for “high nitrogen steels” with a rather low nitrogen content determined by the solubility of nitrogen in the melt (corresponding to the red triangles in Figure 10). For expanded austenite, a different approach must be used. First, there are different Fe-N phases with varying lattice constants, as shown in Figure 10, as well as different references. Secondly, the correct endpoint in this series is the rather metastable phase FeN [34] with the vacancy occupation *x* in FeN_x_ as the variable in the linear extrapolation and not the nitrogen content in at.%.

For expanded austenite MeN_x_ with Me = (Fe, Cr, Ni), there is a saturation limit around *x* ≈ 0.6 [18] beyond where no expanded austenite has been reported. There are data available in the literature which include a series obtained during in situ XRD while sputter etching a gradient layer. Figure 10 aggregates the data in the literature [18,35,36,37] and has been previously published in a different version [36]. For a low nitrogen content, a smaller lattice expansion is observed, including a gap in the lattice constant where no stable phase appears to exist. Beyond that initial region, for 0.15 ≤ *x* ≤ 0.50, there is a region where a linear relation exists between the lattice expansion and the vacancy occupation with nitrogen. For (111), oriented grains of expanded austenite, ref. [18] gives the lattice parameter (in nm) *a* = 0.36396 + 5.9725 × 10^−4^ × *x*. As the lattice parameter for (200) oriented grains is slightly higher, here an offset of 0.37096 nm is used.

**Figure 11:**
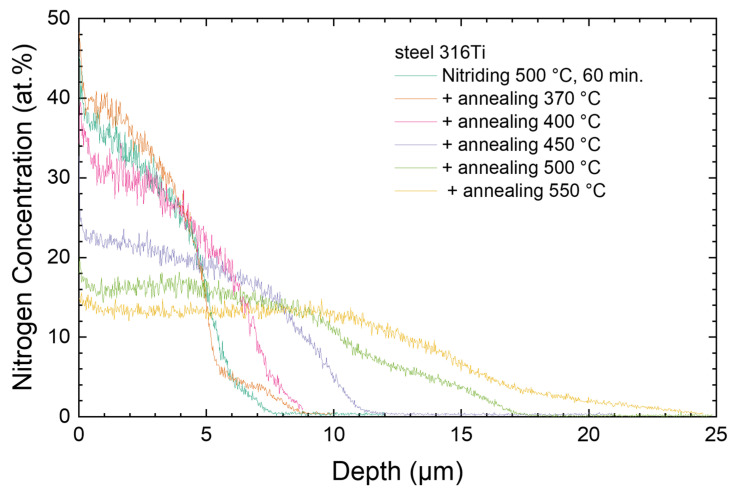
Nitrogen depth profile after nitriding at 500 °C 316Ti for one hour and after additional annealing processes.

While the lattice expansion can be determined in situ during the annealing experiment, the layer thickness can be measured by SIMS only before and after nitriding as no substrate peaks are visible. The summary of the data is shown in Figure 11 for the sample nitrided at 500 °C for 1 h (the data for annealing after nitriding at 450 °C for 1.5 h are quite similar and do not introduce new information). The layer thickness increases from about 5 µm before annealing to values between 5.2 and 18 µm after annealing. There is a significant broadening of the interface for thicker layers due to the increase in the surface roughness for progressive sputtering [25]. This is an artifact not encountered for cross sections of the same samples where an abrupt interface, the respective sharp contrast between two different layers is observed [15,24].

Roughly, a thermally activated nitrogen diffusion is observed for the used annealing temperatures. However, the focus of this manuscript is only on the lattice expansion and not the actual diffusion process. There, an additional investigation of the diffusivity would be interesting, this is albeit outside the current scope as many more samples would be necessary. The point here is that as a first approximation, the total nitrogen content, integrated over the depth, is constant. Hence, for a nitrogen-containing layer with twice the thickness after annealing than before, the occupation of vacancies with nitrogen near the surface (probed by XRD) and throughout the layer should be about 50% of the initial value, e.g., before annealing FeN_0.67_ = 40 at.% nitrogen and after annealing FeN_0.33_ = 25 at.% nitrogen. Thus, the lattice expansion should be similarly reduced by 50% if it is only caused by the insertion of nitrogen. When using the linear extrapolation from Figure 10, care must be taken at very high nitrogen concentrations and respective very high interstitial occupancies. Thus, for the immediate beginning of the annealing, the vacancy occupation derived from the linear approximation of the lattice expansion in Figure 10 can be expected to yield higher values than observed for *x* > 0.50, and the first 1–3 datapoints should be corrected slightly downwards towards a lower nitrogen content.

**Figure 12:**
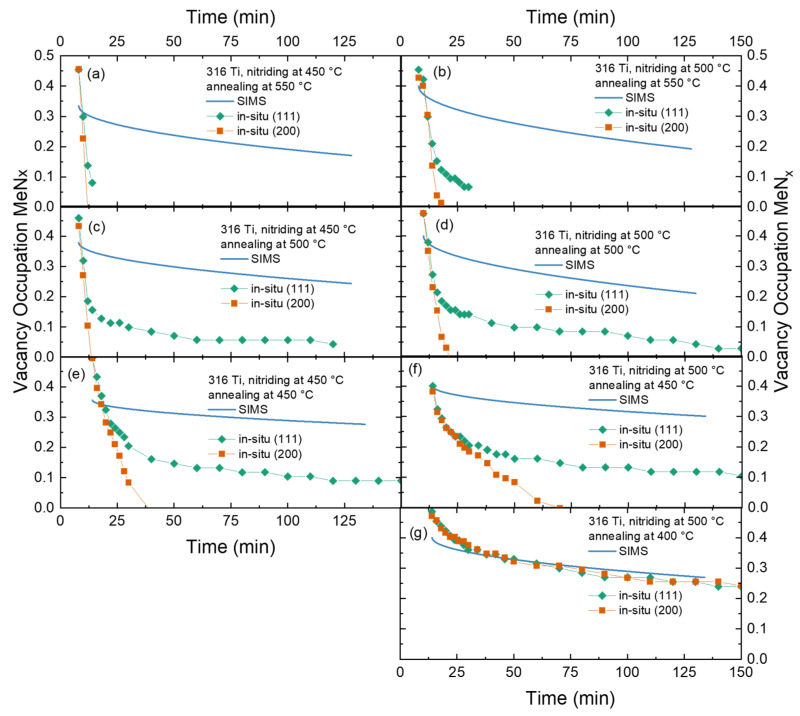
Time series of vacancy occupation with nitrogen during annealing derived from in situ XRD for (111) and (200) oriented grains compared with the interpolation of SIMS data for different annealing temperatures: (**a**) nitriding 450 °C, annealing 550 °C, (**b**) nitriding 500 °C, annealing 550 °C, (**c**) nitriding 450 °C, annealing 500 °C, (**d**) nitriding 500 °C, annealing 500 °C, (**e**) nitriding 450 °C, annealing 450 °C, (**f**) nitriding 500 °C, annealing 450 °C, (**g**) nitriding 500 °C, annealing 400 °C.

In Figure 12, these in situ data from the XRD experiments (cf. Figure 5) of the lattice expansion in the expanded austenite phase, corrected for the thermal lattice expansion and converted in a vacancy occupation, are shown for comparison for different annealing temperatures. Additionally, the temporal evolution of the vacancy occupation derived from SIMS assuming an inverse parabolic layer growth is plotted for comparison as solid lines. The assumption on the growth mode is arbitrary, a linear model or something else could alternatively be used as no details on the actual diffusion as a function of time are known. At the same time, the layers of expanded austenite are too thick to see the substrate as a reference for the layer growth during annealing.

For annealing at 400 °C (cf. Figure 12g), a nearly excellent agreement is observed for both grain orientation and the SIMS data. While the agreement with the inverse parabolic law could be fortuitous, the tendency is very clear. As long as there is no CrN precipitation occurring, and the nitrogen inside the expanded austenite is 100% situated on vacancy positions. Contrasting—or complementing this observation—is the behavior for annealing at 550 °C. There, a complete loss of the expanded austenite is observed, due to CrN precipitation, within the first five minutes. Subsequently, there is a huge discrepancy between the vacancy occupation predicted by SIMS and that from the lattice expansion: 100% of nitrogen is in CrN precipitates. The behavior of the vacancy occupation calculated from the lattice expansion for the (111) oriented grains is less clear than for that derived from the (200) oriented grains as there is some additional background from nanocrystalline material in the XRD data for the former peak position which could be interpreted as remnants of expanded austenite.

For the intermediate temperatures, a situation similar to that shown in Figure 7 is observed. With decreasing temperature, the decay of expanded austenite as inferred here only from the lattice expansion, respective peak position of the expanded austenite (and not from the total phase composition), is becoming slower. Nevertheless, at the end of the annealing experiment at 450 °C and above, a complete transition of the expanded austenite into CrN and a Fe-Ni rich phase is observed. As no additional information about the temporal evolution is accessible from Figure 12, no further analysis of the data towards this goal has been made.

In summary, CrN precipitation in 316Ti is a strongly thermally activated process with the transition starting within less than five minutes at 550 °C and taking more than two hours below 450 °C. During the annealing of samples, the decay of expanded austenite happens throughout the layer simultaneously. While a rather complete and extensive set of experiments has been performed for steel 316Ti to establish the methods for data analysis and understand the results, rather restricted experiments are presented in the following two subsections for the super austenitic steel 904L and the duplex steel 318LN.

### 3.2. Stability of Expanded Austenite in 904L

**Figure 13:**
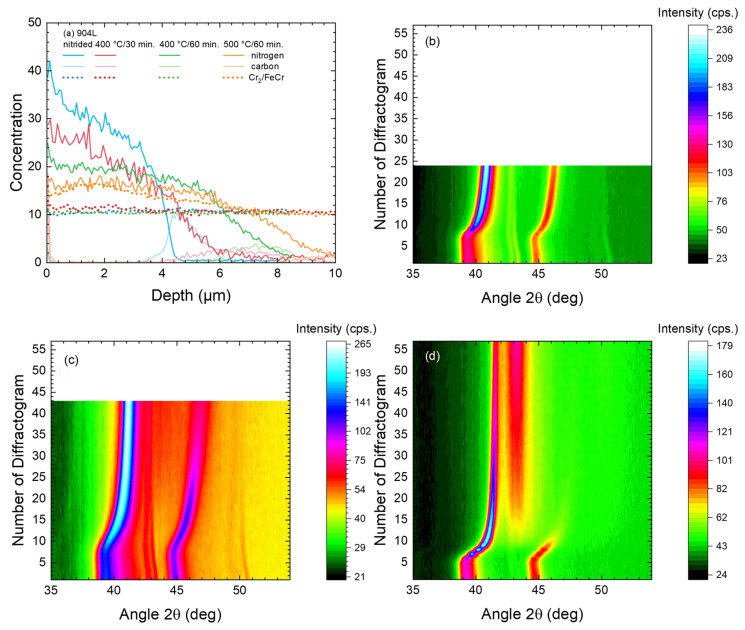
SIMS depth profiles (**a**) and in situ XRD contour plots during annealing of nitrided super austenitic stainless steel 904L: (**b**) 400 °C, 30 min; (**c**) 400 °C, 60 min; (**d**) 500 °C, 90 min.

Steel 904L shows an XRD diffractogram typical for expanded austenite (already plotted above in Figure 3) after nitriding at 450 °C for 90 min with only two broad peaks (identified as expanded austenite (111) and (200)) being visible. For the nitriding process, no large differences between 316Ti and 904L have previously been observed for the nitrogen diffusion during in situ measurement [15]. However, in contrast to 316Ti, 904L tends to form CrN precipitates quite early, which are then apparently pinned at grain boundaries and do not grow considerably during nitriding [15]. However, now we are interested in the thermal stability during annealing as shown in Figure 13.

The data in the SIMS depth profiles (Figure 13a) show a gradual increase in layer thickness after annealing, scaling with time and temperature. At the same time, carbon is again pushed towards the diffusion front. CrN precipitates, indirectly inferred from the Cr_2_^+^/FeCr^+^ cluster ion ratio, are only visible after annealing at 500 °C throughout the whole layer, but are not observed at 400 °C. Complementary information is gained from the contour plots obtained during annealing, including the initial heating phase. For 400 °C, the γ(111) and γ(200) reflections from the underlying substrate decrease with time due to the gradual increase in the layer thickness. Here, an attempt at deriving the layer growth could be made. Yet, the rather low intensities would introduce a larger error without yielding any further information on stability and phase transitions. Nevertheless, the evolution, i.e., the slow decrease, of the lattice expansion is visible for (b) and (c)—which only differ in the total annealing time.

**Figure 14:**
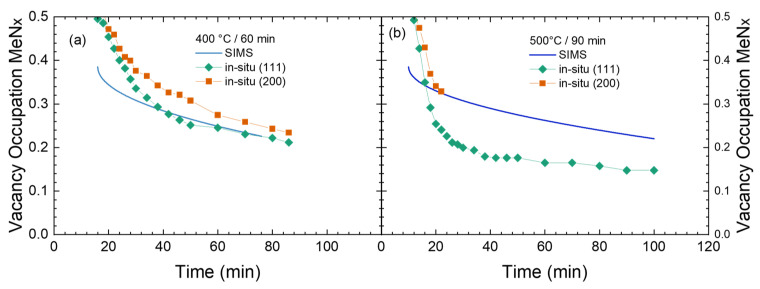
Time series of vacancy occupation with nitrogen during annealing derived from in situ XRD compared with the interpolation of SIMS data for different annealing temperatures of nitrided super austenitic stainless steel 904L. (**a**) annealing at 400 °C for 60 min, (**b**) annealing at 500 °C for 90 min.

In contrast, for 500 °C, the decay of expanded austenite starts again quite early after reaching the final temperature with a concurrent and complete loss of expanded austenite. A comparison with the vacancy occupations is in Figure 14, confirming this result. There, the lattice expansion and the nitrogen content are decreasing slowly in a nearly identical fashion for 400 °C. This indicates, again, the slow decrease in nitrogen caused by nitrogen diffusion towards the bulk. Whereas, for 500 °C, the lattice constant of the expanded austenite is rapidly decreasing after the temperature is reached. After 25 min (including the heating phase of 15 min), γ_N_(200) can no longer be analyzed due to the very low intensity while γ_N_(111) shows again an artifact caused by the overlapping CrN reflection. The rapid decay and the absence of any changes in the peak shape for expanded austenite again point towards a simultaneous start of the decay throughout the whole layer. Thus, decay and diffusion of expanded austenite in super austenitic stainless steel 904L during annealing does not diverge significantly from the behavior observed for austenitic stainless steel 316Ti.

### 3.3. Stability of Expanded Austenite in 318LN

**Figure 15:**
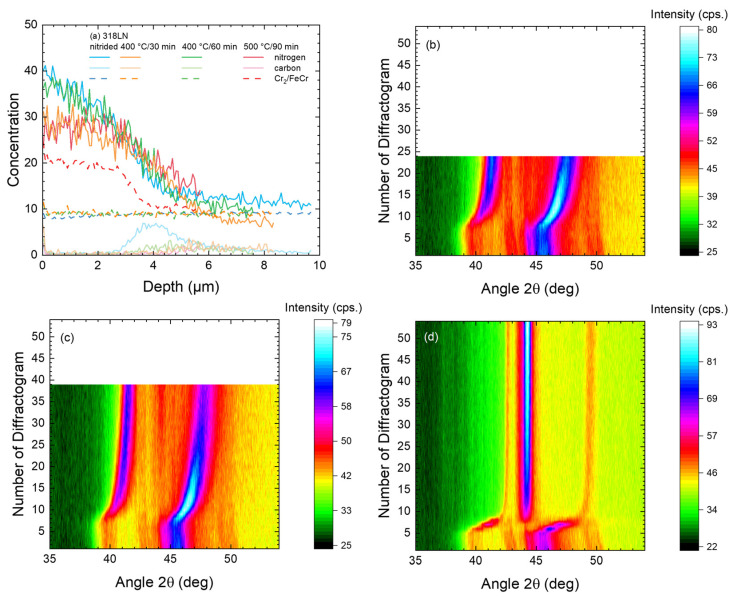
SIMS depth profiles (**a**) and in situ XRD contour plots during annealing of nitrided duplex stainless steel 318LN: (**b**) 400 °C, 30 min; (**c**) 400 °C, 60 min; (**d**) 500 °C, 90 min.

In this last subsection, the annealing behavior of nitrided duplex steel 318LN is investigated using the same annealing conditions as for alloy 904L in the previous subsection. For the sample after nitriding at 450 °C for 90 min, a layered structure of expanded austenite on top of austenitic/ferritic steel is observed (cf. Figure 1 and Figure 3). After annealing, the SIMS depth profiles show only a rather limited diffusion (as shown in Figure 15a) when compared to the diffusion result in the previous two subsections.

**Figure 16:**
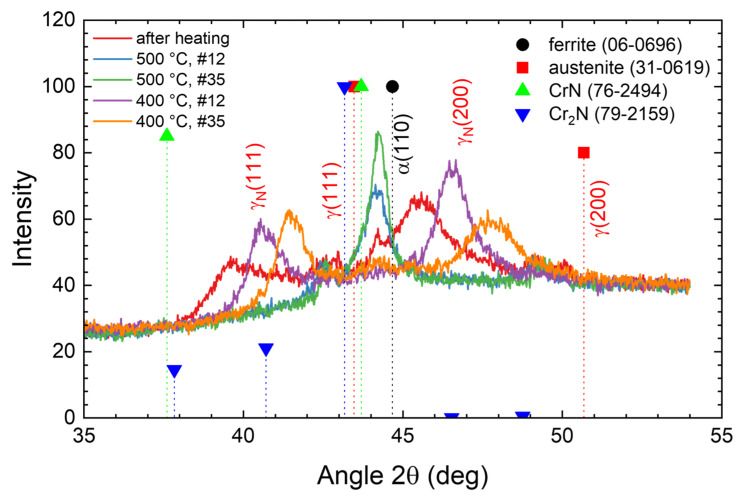
Detailed X-ray diffractograms for nitrided steel 318LN during annealing. Please note that the peak positions from the ICDD Powder Diffraction File Database [38] are measured at room temperature and not at elevated temperatures of 400 and 500 °C, respectively.

For the 500 °C annealing step, a nearly immediate transition towards ferrite and a small hint of CrN is observed in Figure 15d. During the last 88 min of the annealing, barely a change is observed. This result is visible in more detail in Figure 16 where selected diffractograms are plotted for a closer inspection. In contrast, annealing at 400 °C results in a very gradual decrease in the lattice expansion, together with the appearance of a narrow low-intensity peak commonly associated with ferrite or martensite (near 44.5°, shifted slightly towards the left from the literature value for room temperature due to the measurement at 400 °C–or 500 °C). For this reflection, no peak shift but an increase in intensity is observed with time.

**Figure 17:**
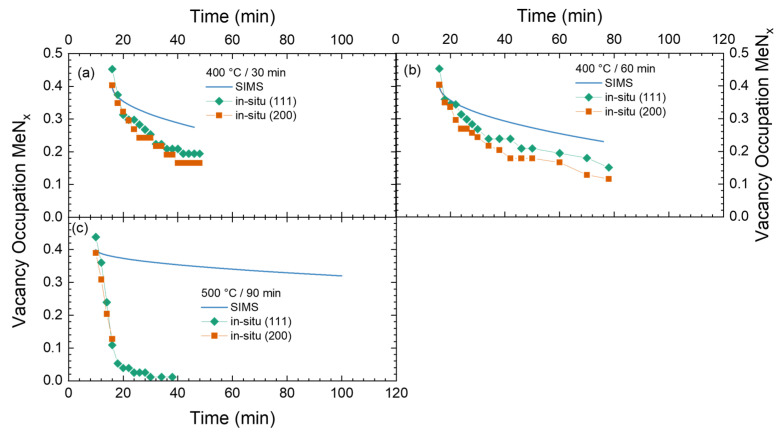
Time series of vacancy occupation with nitrogen during annealing derived from in situ XRD compared with the interpolation of SIMS data for different annealing temperatures of nitrided duplex stainless steel 318LN. (**a**) annealing at 400 °C for 30 min, (**b**) annealing at 400 °C for 60 min., (**c**) annealing at 500 °C for 90 min.

After calculating the vacancy occupation (shown in Figure 17), these tendencies are confirmed. For 500 °C, a very rapid decrease in the lattice expansion is observed, and the diffraction intensity shows the complete decay of expanded austenite and the formation of CrN and a Fe-Ni matrix. However, for this duplex steel, the matrix reverts not to an austenitic structure but (most likely) to a martensitic structure.

For 400 °C, an intermediate behavior is observed with the vacancy occupation dropping faster than expected from the diffusion but not fast enough to indicate a complete decay of the expanded phase. Apparently, segregation into two phases—expanded austenite and martensite (or ferrite) is occurring. Examining the time evolution in detail, no saturation is visible after one hour; yet, the process appears to have slowed down considerably when compared to the initial time dependence.

## 4. Discussion

The presented results for annealing of expanded austenite, without additional nitrogen supply, formed before by nitriding of stainless steel give detailed insight into the stability of this metastable phase. For comparison, only the data in the literature obtained during nitriding, i.e., with a continuous supply of nitrogen from the surface are available. Nevertheless, significant differences—both between the investigated classes of stainless steel and in the behavior with and without nitrogen supply have been observed.

In a previous study [39], it was shown that nitriding/cooling/reheating/nitriding leads to the identical diffusion behavior as a pure nitriding process without interruption. Thus, any relaxation or reordering process occurring during the cooling process (and the subsequent reheating), which takes typically about 5–10 min to go from 400 to 300 °C, does not modify the underlying structure of the expanded austenite. Temperatures below 300 °C are assumed to be uncritical as any thermally activated processes would take longer than the experiment. Actually, in situ XRD yields a constraint on possible atomic rearrangements as there are no significant changes in the diffractograms, except for the thermal shift. Of course, atomic ordering and the annealing of low-dimensional defects are not visible with this method [40].

Returning to the current results, the decay of expanded austenite during annealing in a vacuum without additional nitrogen supply is found to be a thermally activated process with an activation energy of 1.8 ± 0.3 eV. This value is comparable, or slightly higher than for the phase transformation observed during nitriding. At the same time, the time needed to complete the decay is much shorter than during nitriding as the decay starts simultaneously—or nearly simultaneously—throughout the whole layer. In contrast, during nitriding, the nucleation of CrN starts near the surface and consists of a combination of two separate processes—the nucleation and growth of precipitates and the advancement of this transition zone through the expanded austenite from the surface towards the interface with the bulk [29].

Here, this second step is missing for the decay during annealing in a vacuum without additional nitrogen supply. At 500 °C, this transition is already completed after 5–10 min. Thus, the current time resolution of one diffractogram every two minutes—as well as the information depth and the depth resolution—are sufficient to confirm this piece of information unequivocally. However, they do not allow a more detailed description. The other active process during annealing, diffusion of nitrogen driven by the existing concentration gradient appears, using the current preliminary data, to be slower than the diffusion and layer growth during nitriding. While a general concentration-dependent diffusivity [41] can be assumed, details here require further investigation. At the same time, the formation of CrN completely stops the nitrogen mobility as the detrapping of nitrogen—contrary to that from interstitial sites in expanded austenite [30]—is thermodynamically no longer possible. Hence, the nitrogen distribution after the decay of expanded austenite is frozen. Here, further experiments are necessary with a better time resolution where ex situ SIMS must provide the layer thickness after annealing with more data points for intermediate time steps.

While austenitic stainless steel and super austenitic stainless steel show, in general, quite similar behavior during the annealing, regarding both the phase transition into CrN and an austenitic Fe-Ni region and the diffusion, duplex steel is markedly different and necessitates further discussion. Whereas the microstructure consists of austenite and ferrite (with a typical grain size around 2–20 µm, smaller than for the other two steel grades) before nitriding, after nitriding only one homogenous phase of expanded austenite is present. The decay of this phase into CrN and a martensitic Fe-Ni region is plausible from a metallurgical point of view [42]. Yet, the phase composition of the Fe-Ni regions—austenitic or martensitic—is still not investigated in detail. Even for austenitic stainless steel grades, both phases are observed, depending in a yet unknown way on the actual nitriding conditions [39]. However, the slightly surprising result is that for annealing at 400 °C where no CrN is formed for the first 60 min, again a duplex structure of an fcc phase (expanded austenite) and a likely bcc phase (ferrite or martensite) is observed in Figure 16. Here, in situ XRD and SIMS are not sufficient to obtain the complete picture, necessitating additional TEM investigation to explore a possible connection with the known 475 °C embrittlement of ferrite grains in duplex steel where cold working is known to enhance the spinodal decomposition [43].

As during annealing at 400 °C, the temperature is still too low to activate a diffusion of Cr or Ni beyond nearest neighbors, the original chemical composition with a slightly higher Fe/Cr and Ni/Cr ratio in the austenitic than in the ferritic phase must still be present. Apparently, the nitriding introduced just enough nitrogen to allow a transformation of the ferritic phase into expanded austenite. The higher chromium and lower nickel content of duplex steels is adjusted to allow a partial transformation during cooling from ferrite to austenite for some parts of the steel while small chemical gradients are established within the material [42].

Yet, during annealing, this transition appears to have been reversed. As the solubility of nitrogen in martensite (and ferrite) is much lower than for austenite, excess nitrogen beyond the solubility should then be stored along grain boundaries and defects. That behavior has been documented for nitriding of ferritic stainless steels [44,45]. These are at a different position in the Schäffler diagram [46]; thus, a conversion into austenite with sufficient nitrogen uptake during nitriding was not observed in these steels where a stable ferritic phase is established. At the same time, 475 °C embrittlement could also be occurring even at 400 °C, at a lower rate. Without nitrogen, a spinodal decomposition of the super-saturated solid ferrite solution into an Fe-rich and a Cr- nanophase can happen [43,47]. Similar processes may be active here during annealing. Furthermore, nitrogen diffusion requires some mobility [30]. Thus, after CrN formation, the nitrogen distribution is frozen, and the depth profile measured by SIMS only reflects the diffusion within expanded austenite before any phase transition. Similarly, the observed changes during annealing at 400 °C may lead to reduced mobility and thus no nitrogen redistribution even without CrN precipitates.

The current results are complementary to a recent publication where nitrided super duplex steel was annealed in air and investigated by in situ synchrotron radiation [48]. There, nitriding at 350 °C led to the simultaneous formation of expanded austenite and expanded ferrite. As the steel used there, X2CrNiMoN25-7-4 has a slightly higher Cr content; it is plausible that the transition from ferrite to austenite during nitriding is not observed as the position of this steel in the Schäffler diagram is slightly different [46]. They also observe a combination of thermal expansion and reduced lattice constant during heating as nitrogen diffusion reduces the local surface concentration. Simultaneously, the surface hardness decreases from around 15 to 13 GPa after annealing at 450 °C). Finally, at 550 °C chromium nitride and iron oxides are observed.

However, at the current stage of the investigations, any details of microscopic atomic rearrangements observed during annealing at lower temperatures are not accessible from XRD and SIMS alone. Hence, additional TEM investigations are advised to elucidate more details. Similarly, thermal activation for longer annealing times and at higher temperatures is advised. Here, information on the actual nucleation sites where CrN formation starts is required. Conversely, details of the metallurgical stability of austenite and the transition from ferrite to austenite and back to ferrite for the high nitrogen regime are urgently needed to understand the secondary phase transitions—especially for duplex (and super duplex) stainless steel.

While the thermally activated decay of expanded austenite into CrN during annealing does not present fundamentally new information, the nitrogen diffusion in austenite/super austenite and the phase transitions accompanying this diffusion in duplex steel need additional attention in a follow-up investigation for conditions where no CrN is formed.

## 5. Conclusions

In situ XRD together with cluster analysis in SIMS is a powerful tool to establish information on diffusion and phase formation as well as the local neighborhood of atoms when using information from molecular ions even for inorganic solids. The current investigation yields a rich data set while using only a limited experiment. Here, the annealing of expanded austenite in a vacuum without any additional supply of nitrogen was investigated.

Thus, the CrN nucleation, precipitation, and growth of precipitates was observed independent from the macroscopic movement of this transition front from the surface through the expanded austenite layer towards the substrate. During nitriding experiments at higher temperatures, both effects are observed simultaneously, and the published activation energy there actually comprises both effects.

Extrapolating the current results towards lower temperatures, the very high observed activation energy for the decay into CrN should allow the operation of expanded austenite layers up to 350 °C. Here, though, the limitation is given by the diffusion of nitrogen towards the bulk, thus reducing the surface hardness and, correspondingly, the wear resistance and increasing the cold-welding tendencies on a timescale of 10,000 h. For lower temperatures, much longer operation times are possible.

## Figures and Tables

**Table 1:** Summary of nitriding conditions used for the samples before annealing in vacuum.

Alloy	Nitriding Temperature	Nitriding Time
316Ti	450 °C	90 min.
316Ti	500 °C	60 min.
904L	450 °C	60 min.
318LN	450 °C	60 min.

## Data Availability

The raw data supporting the conclusions of this article will be made available by the authors upon request due to privacy.
